# Non-Invasive Neurostimulation Methods for Acute and Preventive Migraine Treatment—A Narrative Review

**DOI:** 10.3390/jcm10153302

**Published:** 2021-07-27

**Authors:** Stefan Evers

**Affiliations:** 1 Faculty of Medicine, University of Münster, 48153 Münster, Germany; everss@uni-muenster.de; 2 Department of Neurology, Lindenbrunn Hospital, 31863 Coppenbrügge, Germany

**Keywords:** neurostimulation, vagal nerve, supraorbital nerve, transcranial magnetic stimulation

## Abstract

Neurostimulation methods have now been studied for more than 20 years in migraine treatment. They can be divided into invasive and non-invasive methods. In this narrative review, the non-invasive methods are presented. The most commonly studied and used methods are vagal nerve stimulation, electric peripheral nerve stimulation, transcranial magnetic stimulation, and transcranial direct current stimulation. Other stimulation techniques, including mechanical stimulation, play only a minor role. Nearly all methods have been studied for acute attack treatment and for the prophylactic treatment of migraine. The evidence of efficacy is poor for most procedures, since no stimulation device is based on consistently positive, blinded, controlled trials with a sufficient number of patients. In addition, most studies on these devices enrolled patients who did not respond sufficiently to oral drug treatment, and so the role of neurostimulation in an average population of migraine patients is unknown. In the future, it is very important to conduct large, properly blinded and controlled trials performed by independent researchers. Otherwise, neurostimulation methods will only play a very minor role in the treatment of migraine.

## 1. Introduction

One of the recent innovations in migraine treatment was the detection of several types of neurostimulation for acute and preventive treatment [1]. The basic idea behind this is the stimulation of peripheral and cranial nerve structures or of the cortex to reduce migraine pain or to decrease migraine frequency. This follows a general development in pain therapy showing that specific stimulations of specific nervous structures can lead to a decrease in pain.

In this narrative review article, the non-invasive neurostimulation methods that have been studied in migraine treatment are presented. Non-invasive in this context means that electrical or other stimulation is applied without penetrating the skin by any means. It is also called external stimulation. Neurostimulation in this context means that nerve fibers are stimulated by electric, magnetic, thermal, or mechanic stimulation; it is also called neuromodulation, since the stimulation of peripheral nerve structures is probably not the basic mechanism for pain/headache relief, but leads to a modulation of central pain processing structures. This review focusses on migraine. This means that both major types of migraine (episodic and chronic) are considered as migraine; many clinical trials differentiated between the treatment of episodic migraine and the treatment of chronic migraine and between acute and prophylactic migraine treatment. Invasive neuromodulation procedures were excluded from this review.

It is not the purpose of this review to speculate on the mechanisms by which neuromodulation can have an impact on migraine. The basic idea is that migraine is a disorder of neuronal networks, and that it is possible to modulate this network by different mechanisms and in different neuroanatomical sites. In particular, presynaptic neurotransmitter release can be influenced by external stimuli; this can lead to inhibitory or excitatory processes in cortical structures and/or in brainstem structures which are involved in migraine pathophysiology. Different patient types, different headache types, and different situations (acute versus prophylactic treatment) might be the factors causing the different efficacies of neuromodulation.

The majority of clinical trials on neuromodulation in migraine have been performed in refractory patients. This means that we do not really know the efficacy and applicability of these methods in the general migraine population, but only for the severely handicapped patients. This was made possible because the European CE mark and FDA regulatory requirements for medical devices are relatively easier to achieve than approval for pharmaceutical and biological therapies.

Non-invasive neurostimulation for the treatment of migraine has become quite popular in recent years, since it is an effective, minimal-risk option for some patients. The first study of neurostimulation in headache disorders involved invasively implanted electrodes for intractable occipital neuralgia; several implantable and non-invasive devices have since been studied in clinical trials.

The methods comprise non-invasive transcutaneous vagal nerve stimulation (tVNS), transcutaneous electrical nerve stimulation of different nerves (tNS), single or repetitive transcranial magnetic stimulation (TMS), and transcranial direct current stimulation (tDCS) [2]; in addition, thermic and mechanic stimulation is also included.

## 2. Neurostimulation Methods

### 2.1. Transcutaneous Vagal Nerve Stimulation

Non-invasive transcutaneous vagal nerve stimulation (tVNS) is a device applying electrical stimulation to the part of the neck in which the area can be found where the vagal nerve descends. The vagal nerve contains the majority of parasympathetic nerve fibers for the rest of the body. Stimulation of the vagal nerve can modulate neuronal activity probably involved in the pathophysiology of migraine (and other disorders such as epilepsy and depression), including the locus coeruleus, nucleus tractus solitarious, and trigeminal spinal tract [3].

In an open pilot study, this method was efficacious in the treatment of acute migraine attacks [4]. However, controlled studies replicating this finding are missing. The non-invasive Vagus Nerve Stimulation as Acute Therapy for Migraine (PRESTO) trial [5] examined patients with migraine using tVNS versus sham treatment as an acute therapy of migraine attacks (using the gammaCore^TM^ device). The primary endpoint (pain freedom at 2 h) was not met, but some secondary endpoints were statistically significant in favor of tVNS, including pain freedom at 0.5 and 1 h. A post hoc analysis of this study showed that tVNS also led to decreased intake of rescue medication and that those participants with a mild headache at onset reached statistically significant pain freedom at 2 h.

Many more trials were performed on the prophylactic treatment of migraine with tVNS. The first observation on four patients with severe chronic migraine responding to tVNS, with a decrease in headache days, was published in 2009 [6].

The EVENT study with 59 patients with chronic migraine randomized with regard to using tVNS versus sham treatment did not show relevant significant differences between the intervention and control groups with respect to migraine prophylaxis [7]. Additionally, another trial on the prophylaxis of migraine, the PREMIUM trial, did not show a statistically significant reduction in migraine days (primary endpoint) when comparing tVNS to sham in patients with episodic migraine. Significant benefits were only seen in a post hoc analysis of participants who were particularly compliant with the treatment [8]. The most recent study published on the efficacy of tVNS in migraine prophylaxis [9] also showed no significant efficacy of tVNS. A pooled analysis of these three randomized trials [7,8,9] showed an absence of heterogeneity but did not demonstrate a significant efficacy of tVNS in migraine day reduction (0.187, 95% CI: 0.379 to 0.004) [10].

The tVNS as applied by the gammaCore^TM^ device in the prophylaxis of migraine consists of two stimulations (each 2 min long) thrice a day. For acute attacks, treatment consists of two stimulations applied at headache onset, followed by two more after 20 min and after 2 h if needed. Side effects include stiff neck, frequent urination, lip/facial droop, mild confusion, and dizziness. Contraindications include active devices near the site of stimulation (such as pacemakers, defibrillators), as well as carotid atherosclerosis, history of cervical vagotomy, significant hypertension, hypotension, bradycardia, or tachycardia.

A small study investigated the efficacy, safety, and tolerability of the stimulation of an auricular branch of the vagal nerve [11]. The stimulation was applied for 4 h per day. Those patients stimulated with a frequency of 1 Hz showed a significantly higher reduction in headache days per month than those patients stimulated with 25 Hz (−7.0 ± 4.6 versus −3.3 ± 5.4 days, *p* = 0.035). However, there was no sham control and there is no replication study on this type of vagal nerve stimulation.

Although the evidence for the acute treatment of migraine attacks by tVNS is poor and the evidence for prophylactic treatment is negative, the device received FDA approval for both acute and prophylactic treatment in the USA.

### 2.2. Transcutaneous Nerve Stimulation

Different types of transcutaneous nerve stimulation (tNS) were tested in migraine treatment. The principle is always the same: applying an electrode over a peripheral (cranial) nerve and daily stimulation with different intensity and frequency. The bilateral transcutaneous stimulation of the supraorbital nerve (which in reality often includes in part also of the supratrochlear nerve) has been studied most often [12]. In a sham-controlled study, 67 patients were included [13]. After 3 months, the number of migraine attacks was significantly reduced by the verum stimulation (6.94 versus 4.88; *p* = 0.023) in contrast to sham stimulation (6.54 versus 6.22; *p* = 0.608). The 50% response rate was significantly higher after verum stimulation (38.1%) than after sham stimulation (12.1%). In two open trials on patients with episodic migraine and on patients with chronic migraine, 75% and 50%, respectively, of the patients reached a significant reduction in days with acute analgesic/triptan intake or with headache [14,15].

Another study examined the simultaneous use of three tNS devices to stimulate the face, the cervico-occipital region, and the hand. The stimulation was used 5 days/week for three consecutive weeks and was compared to laser therapy and to acupuncture [16]. Only qualitative analysis was performed. After one month, tNS and laser therapy where more efficacious than acupuncture. Two trials examined the use of occipital tNS as a prophylactic therapy for migraine. One of them used 40 Hz stimulation and was negative [17]. Various stimulation parameters were used for the other one, and 100 Hz was positive as compared to sham but less effective than topiramate [18]. Recently, one study combined the oral migraine prophylactic drug flunarizine with supraorbital nerve stimulation, and a significant additional effect of the stimulation was shown as compared to sham stimulation [19]. In another study on patients not tolerating or refractory to topiramate for the prophylaxis of chronic migraine, tNS of the supraorbital nerve resulted in a decline in headache days over 3 months [20].

The results of the trials on migraine prophylaxis were also reflected in a survey on 2313 patients having used the Cefaly^TM^ device [21]. After a testing period of about 60 days, 46.6% of the patients were not satisfied and returned the device, and the compliance check showed that they used it for 48.6% of the recommended time. Overall, 54.4% of the patients were satisfied with the device. Meanwhile, 4.3% reported one or more adverse event (particularly paresthesia and local pain).

The Acute Treatment of Migraine with External Trigeminal Nerve Stimulation (ACME) trial examined the acute use of the Cefaly^TM^ device in episodic migraine patients [22]. This was a randomized, double-blind, sham-controlled trial with 106 patients showing improvement in the primary outcome (change in pain score at 1 h compared to baseline) with a significant result in both the treatment and the sham group, although the findings were more significant for the verum group (59% versus 30%, *p* < 0.0001). Pain measurements using the visual analog scale (VAS) also showed statistically significant differences between the treatment and the sham group at 1, 2, and 24 h. A recent study replicated this finding in the emergency room, with a significant effect of supraorbital nerve stimulation in acute migraine attacks [23].

Applying the Cefaly^TM^ device consists typically of a 60 min session for acute therapy and 20 min daily for prophylactic use. This method is typically well-tolerated. Side effects include forehead paresthesia (in particular when used outside an acute migraine attack), sleepiness, fatigue, insomnia, and headache. Contraindications for its use include recent facial trauma, metallic head implants, or intracardiac lines or pacemaker devices. Cefaly^TM^ received FDA approval for both acute and prophylactic treatment.

Even a combination of two different eNT (Relivion^TM^) has been studied. Patients received the combination of external occipital nerve and supraorbital nerve stimulation for the treatment of migraine [24]. However, only review data on the efficacy of this device have been published so far, and no primary trial results could be found in the literature, although this device received FDA approval.

A single double-blind study showed mild efficacy by stimulating the mastoid region percutaneously [25]. Eighty patients with episodic migraine were included. In the verum group, 82.5% were 50%-responders, whereas, in the sham group, only 17.5% responded. The same group recently compared supraorbital tNS versus tNS of the mastoid region and observed a statistically significant reduction in migraine days in the third month in both groups [26]. The difference between the two groups was not significant (77.8% responders in the mastoid group and 62.2% in the supraorbital nerve group).

### 2.3. Magnetic Stimulation

#### 2.3.1. Acute Attack Treatment

Probably the first scientific study on the impact of magnet fields on headache was published in 1985 [27]. Forty patients with different types of headache were treated with alternating pulsed magnetic field stimulation or with a sham stimulation, both around the whole head. An improvement of headache was reported by the majority of patients after verum stimulation but not after sham stimulation. Interestingly, the results were better for tension-type headache than for migraine. However, this study did not apply a regional magnetic impulse but a global magnetic field. Another study on different headache types observed similar results when a global magnetic field was used [28]. Since this time, different methods have been tested to treat migraine and headache by magnetic stimulation. These include single pulse transcranial magnetic stimulation (sTMS) and the repetitive transcranial magnetic stimulation (rTMS), in addition to peripheral nerve magnetic stimulation.

The idea behind magnetic stimulation to treat migraine attacks is influencing the cortical excitability by the magnetic impulse and thus stopping the migraine aura and the subsequent headache. In animal studies, it has been shown that a single magnetic impulse is able to stop the cortical spreading depression [29]. Two studies showed the good efficacy of sTMS in the acute treatment of migraine attacks with an aura [30,31].

In the first (uncontrolled) study, 42 patients were treated by two sTMS impulses over the painful skull (migraine without an aura) or over the occipital cortex (migraine with an aura) [30]. A reduction in headache intensity was observed both after low and after high stimulation intensity, and 32% of the patients reported complete headache abortion for 24 h. All patients with an aura reported a sudden effect on the headache. This observation led to the second study [31] which randomized 164 patients with migraine with aura. Migraine attacks were treated using a hand-held magnetic stimulation device with two impulses over the occipital cortex within one hour after onset of the aura. The study was sham-controlled. The responder rate for being pain-free after 2 h was significantly higher after verum treatment (39% for sTMS versus 22% for sham), as was being persistently pain free after 24 and 48 h. However, the global success of the treatment was rated better for the sham than for the verum stimulation. The device received FDA approval (sTMS mini^TM^). A post-market pilot study performed in the United Kingdom in 2015 reported that 62% of patients experienced pain relief and a reduction in monthly headache days in both episodic and chronic migraine [32].

The methodological problems of these studies are the poor sham control, since the magnetic impulse led to an unpleasant feeling, whereas the sham impulse did not. This type of migraine attack treatment has only been proven in migraine with aura patients, which represent only up to 30% of all migraine patients; in addition, not all aura patients have an aura every time they have a migraine attack.

#### 2.3.2. Prophylactic Treatment

The first anecdotal reports on the efficacy of rTMS in migraine was published in a study on rTMS in major depression. Two patients, blinded for the treatment with rTMS, reported a disappearance of their migraine during the study phase [33].

In addition, controlled trials have been performed on the prophylaxis of migraine by rTMS. In one study, the left dorsolateral prefrontal cortex (DLPFC) was stimulated with 20 Hz rTMS. Attack frequency, headache index, and the number of acute medications were significantly reduced in six patients with chronic migraine as compared to five patients receiving sham stimulation [34]. Another study on 13 patients with chronic migraine applied 10 Hz rTMS and was unable to replicate the results of the first study, and rTMS was even less effective than sham stimulation [35]. In a further study, the cortical hyperexcitability in chronic migraine was lowered by 1 Hz rTMS over the vertex [36]. The frequency of migraine attacks was, however, not significantly reduced by the verum stimulation as compared to the sham stimulation.

Three studies (two conducted by the same group) used high frequency (10 Hz, 600 pulses) rTMS over the left primary motor cortex (M1) for the prophylaxis of chronic migraine. A single session reduced the number of headache days per month for chronic migraine sufferers by an absolute number of 3.2 days/month versus placebo [37]. In total, 98% of the patients had a more than 50% reduction in headache frequency after 2 weeks, and this improvement persisted until week 4 in 80.4% of the patients. Pain intensity, functional disability, and acute drug intake were reduced during the total study time; the best result, however, was obtained for the first 2 weeks. Later, the second study showed that three sessions did not provide better pain reduction than a single session of rTMS [38]. One study compared botulinum toxin A injection with high frequency rTMS (10 Hz, 2000 pulses per session) over the left M1 with a total follow-up of 12 weeks on patients with chronic migraine [39]. As compared to botulinum toxin A, rTMS showed no difference at weeks 4 and 8, but was less effective at week 12. The pooled analysis of these three studies focusing on high frequency rTMS over the left M1 suggested a positive effect, with a medium effect size of −0.533 (95% CI −0.940 to −0.126) [10].

The ESPOUSE study, a multicenter, open-label, observational study including 132 episodic and chronic migraine patients, found a mean reduction of −2.75 headache days from baseline versus placebo with −0.63 headache days (*p* < 0.0001) over a 3-month period of rTMS treatment [40].

Two other recent studies with stimulation of the left DLPFC were positive in the primary outcome, using a frequency of 5 Hz in one case [41] and intermittent theta-burst stimulation in the other [42]. However, the first study was negative for the reduction in headache days. The pooled analysis of these two studies focusing on high-frequency rTMS over the left DLPFC did not favor a positive effect and showed high heterogeneity between studies [10].

The safety and tolerability of TMS in migraine treatment was shown in a review [43]. Contraindications for its use include seizure as well as conductive implants and materials in the head and upper body, such as pacemakers and vagal nerve stimulators. Interestingly, a prophylactic oral medication seems to have no influence on the efficacy of rTMS, and patients without prophylactic medication showed a better response to sham than those with prophylactic medication [44].

#### 2.3.3. Peripheral Magnetic Stimulation 

Magnetic stimulation has also been tried outside the head to treat migraine. One study used peripheral pulsed electromagnetic fields applied to the wrist and was negative [45]. Another study tested repetitive magnetic stimulation to myofascial trigger points of the neck compared to shoulder muscle stimulation [46]. There was no difference between the two stimulation sites, although a reduction relative to baseline was seen in both groups.

### 2.4. Transcranial Direct Current Stimulation

Transcranial direct current stimulation (tDCS) in a migraine attack was first studied in 2011 in 62 patients in a controlled study [47]. Both the tDCS and the sham stimulation resulted in a reduction in headache intensity by 54.2%. The authors interpreted this as an unspecific effect of tDCS.

The effects of a cathodal tDCS versus sham stimulation (three times a week over the primary visual cortex) were studied in 26 patients with episodic migraine, assuming that the cortex is hyperexcitable between the attacks [48]. The number of migraine days, attack duration, and attack intensity improved significantly as compared to baseline but not as much compared to sham stimulation (except for the attack intensity). Side effects were mild and transient. Based on the contrary assumption, another study on 13 patients with chronic migraine applied anodal tDCS over the primary motor cortex [49]. A delayed effect on pain intensity and attack duration was observed 120 days later; this was regarded as a slow modulation of the cerebral pain matrix.

One study investigated the use of left M1 anodal tDCS for 20 consecutive days com-pared to sham stimulation and was positive, with an absolute difference of 1 day per month [50]. Another study also investigated anodal tDCS over the dominant M1 and was positive after 10 sessions applied over 30 days [51]. A further study compared cathodal tDCS over the right M1 area or the primary sensory cortex (S1) versus sham stimulation for 22 sessions over 10 consecutive weeks. The two active stimulation groups were effective [52]. The difference was very large in both groups, with 12.5 days/month for the M1 versus 9.5 for the S1 group compared to the sham group. Another recent study compared anodal, cathodal, and sham tDCS over the right M1, used daily for the 5 days of withdrawal treatment for patients with chronic migraine associated with medication overuse [53]. There was no significant difference in the number of migraine days per month 12 months later.

Another recent study examined the use of five daily sessions of cathodal tDCS over the coolest frontal region and showed efficacy for the reduction in migraine days, with a large effect size and an absolute reduction by about 8 days/month relative to the placebo group [54].

The hypothesis that self-administered anodal tDCS over the visual cortex significantly decreases the number of monthly migraine days in episodic migraine was tested in an observational study on 26 patients. The overall result was that patients had a significantly lower number of monthly migraine days at follow-up after verum versus sham stimulation. However, the stimulation had neither an immediate nor a long-term effect [55].

### 2.5. Remote Electrical Neuromodulation

Remote electrical neuromodulation (REN) is a more recent method of neurostimulation in primary headache disorders. This treatment provides neural stimulation to the nerves in the upper arm, which is assumed to modulate pain by activating conditioned pain modulation. A prospective, double-blind, randomized, crossover, sham-controlled trial published in 2017 using the Nerivio Migra^TM^ device to abort acute migraine attacks studied 71 patients and found a 50% pain reduction in 64% of participants with migraine versus 26% for sham stimulation [56]. In 2019, a randomized, double-blind, multicenter placebo-controlled trial in 296 participants using REN found that two thirds of patients achieved pain relief versus 38.8% using sham (*p* < 0.0001) [57]. The device was approved by the FDA for the acute treatment of migraine with or without aura in patients 18 years and older. The device is worn on the upper arm and provides 45 min of stimulation per treatment, controlled by a smartphone application. By this, the patient can control the level of intensity; the patient is instructed to adjust the intensity to the strongest stimulation level under the pain threshold. Adverse events with REN are not common, although some reported side effects including a warm sensation, arm/hand numbness, itching, pain, tingling, and muscle spasms. Contraindications for use include congestive heart failure, uncontrolled epilepsy, and active implantable medical device (pacemakers, hearing aid implant, etc.)

The efficacy in adults has induced an open trial in children which was recently finished and suggested an efficacy also in children and adolescents [58].

### 2.6. Non-Electrical Stimulation

All the stimulation techniques discussed so far in this review were based on direct electrical stimulation or on indirect electrical stimulation via magnetic fields. Other stimulation techniques use mechanic or temperature-based stimulations in the treatment of migraine.

Caloric vestibular stimulation (CVS) is a method of stimulating the ear by frequent periodic temperature changes via a specific device similar to an earphone (TNM^TM^). One randomized and sham controlled trial is available with 100 patients with episodic migraine [59]. After 3 months of treatment, the verum group exhibited significantly fewer migraine days (−3.9 ± 0.6) than the sham group (−1.1 ± 0.6). No serious adverse events were reported. The device was well-tolerated. Further studies on CVS in migraine treatment cannot be found in the literature.

Kinetic oscillation stimulation (KOS) is a technique with high frequency oscillating pressure on the nasal mucosa administered by an intranasal plastic catheter. Two small studies, one of them controlled, from the same group showed the efficacy of this stimulation technique in acute migraine attack reduction [60,61].

## 3. Discussion

Based on this analysis, one can conclude that most devices are safe and show some efficacy in single trials and that both magnetic and electrical stimulation techniques might have a place in migraine treatment with moderate efficacy and with few adverse events. However, it is the general impression that these non-invasive devices might show less efficacy than invasive stimulation techniques such as the sphenopalatine ganglion stimulation or the occipital nerve stimulation. In Table 1, the devices are summarized according to the personal judgement of the author. The best evidence for acute migraine attack treatment has been shown for eNS of the supraorbital nerve (in all migraine types) and for sTMS (for migraine with aura); to a lesser extent, REN has also shown efficacy in acute migraine attack treatment. In the prophylaxis of migraine, only eNS of the supraorbital nerve has shown convincing efficacy. For the other stimulation techniques, conflicting results (or no studies) have been published.

Furthermore, there is some evidence that the devices are more efficacious in chronic migraine than in episodic migraine. This might be, however, a bias, since most of the studies were performed on chronic migraine and only few studies reported on refractory episodic migraine. We simply do not know the efficacy of these devices in a “normal” episodic migraine. With respect to other migraine subtypes, no comparative studies have been performed.

Many patients accept these devices even if they are less effective than oral drug prophylaxis because they fear the side effects of oral drugs. On the other hand, several patients do not respond to any device at all. The approval of these devices in the USA and in Europe is different and the reimbursement situation in single countries is not easy to understand.

The role of the devices in migraine treatment is still and will remain, even in the coming years, unclear. After the first few years, characterized by enthusiasm, real-world experience has shown that the efficacy of many devices is not as good as had been expected from the clinical trials. Furthermore, there have been problems in the manufacturing, approval, and availability of some devices. 

The unclear picture of the efficacy of the devices is also based on the complex treatment situation in migraine therapy. Many devices have been tested for both acute and prophylactic treatment but only shown probable efficacy in one of the two situations. Most of the clinical trials separated episodic and chronic migraine patients, but some included all types of migraine patients. For many devices, different stimulation parameters have been tested, meaning that many studies are not comparable; the same is true for the site of stimulation. 

Given the heterogeneity of study results for all devices, it is a little surprising that nearly all devices have received FDA approval and that some devices also received EMA approval (CE mark). The experience resulted from this situation is, however, low and not fully published, meaning that real world data are missing.

It is doubtful whether these devices will gain more evidence for their efficacy and will improve their place in migraine treatment protocols in the future; to do so, large, controlled trials addressing either acute or prophylactic treatment need to be performed, combining other treatments and, preferably, without a commercial sponsor. Given the recent progress in the drug treatment of migraine by small molecules for acute attack treatment and by the CGRP antibodies for the prophylaxis of migraine, one might remain skeptical about the future of medical devices in the treatment of migraine as long as clinical research does not support their efficacy in future studies.

In the future, however, there may arise new approaches including closed loop stimulation [62]. However, the problem with closed loop or individualized stimulation is the lack of specified treatment targets, in particular for pain disorders. This may change with the detection of specific treatment targets by fMRI or TMS-EEG.

## 4. Disclosures

The author has received honoraria from Allergan/Abbvie, Lilly, Lundbeck, Novartis, and Teva for advising and/or speaking in the past three years. He has no connections to any companies manufacturing neurostimulation devices.

## Figures and Tables

**Table 1:** Different types of non-invasive neurostimulation in the acute and prophylactic treatment of migraine with the probability of efficacy.

Method	Device	Acute Treatment	Prophylactic Treatment
tVNS	gammaCore^TM^	no evidence of efficacy	no evidence of efficacy ^1^
eNS	Cefaly^TM^	probably effective	probably effective ^1^
sTMS	sTMS mini^TM^	probably effective ^2^	not studied
rTMS	-	not studied	conflicting evidence of efficacy
tDCS	-	not studied	conflicting evidence of efficacy
REN	Nerivio Migra^TM^	probably effective	not studied
CVS	TNM^TM^	not studied	possibly effective
KOS	-	possibly effective	not studied

^1^ only episodic migraine, ^2^ only migraine with aura. tVNS = transcutaneous vagal nerve stimulation; eNS = electrical nerve stimulation; sTMS = single transcranial magnetic stimulation; rTMS = repetitive transcranial magnetic stimulation; tDCS = transcranial direct current stimulation; REN = remote electrical neuromodulation; CVS = caloric vestibular stimulation; KOS = kinetic oscillation stimulation.

## Data Availability

Not applicable.
